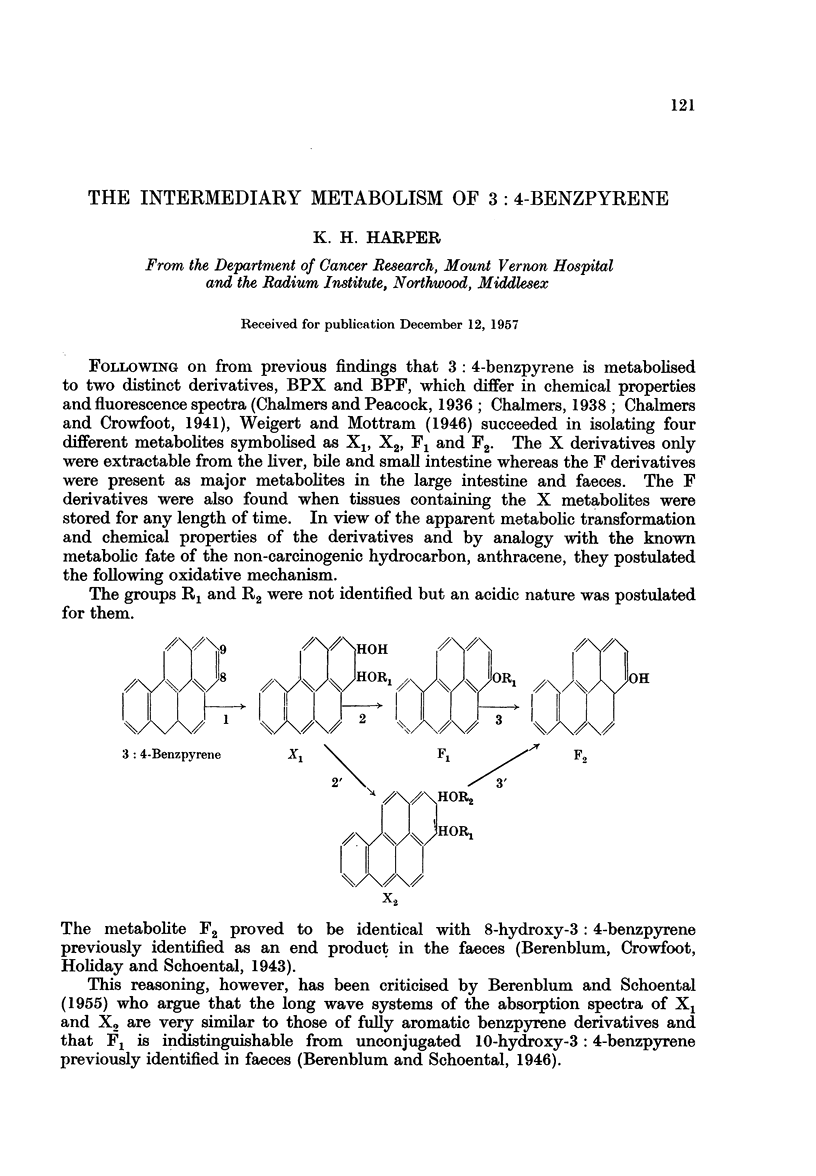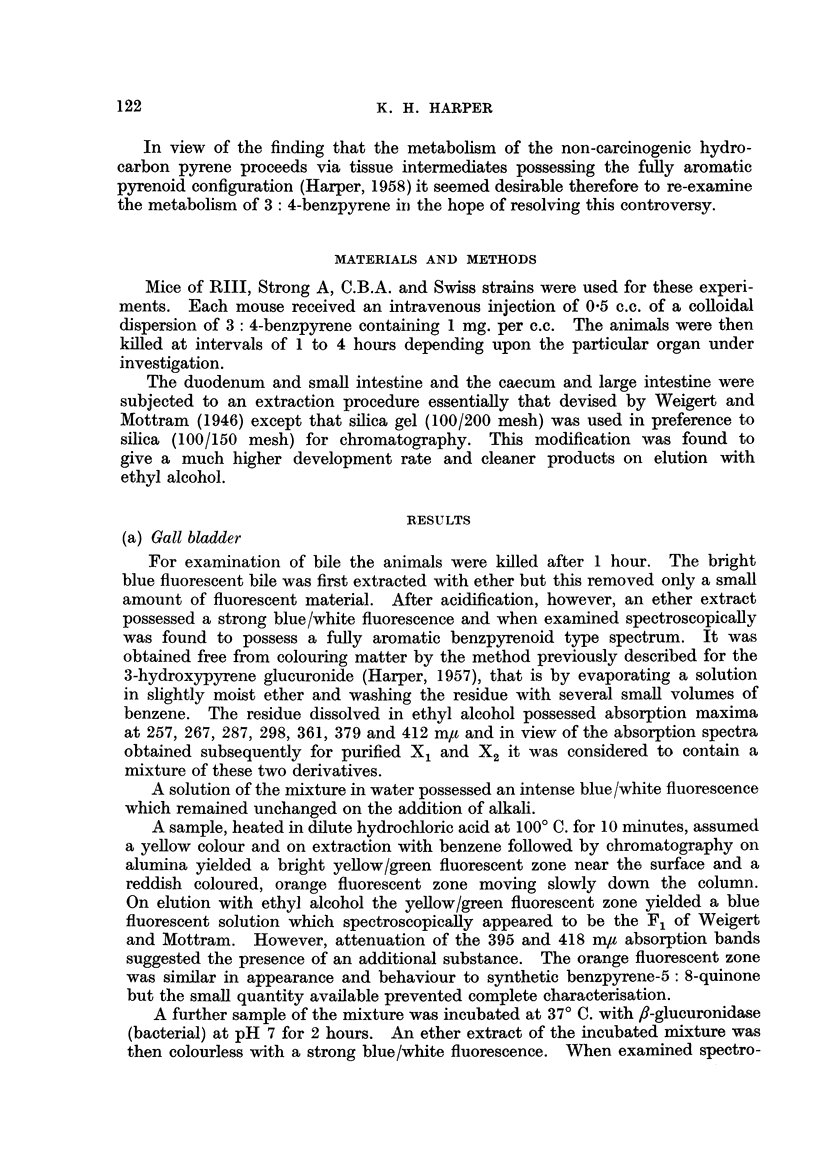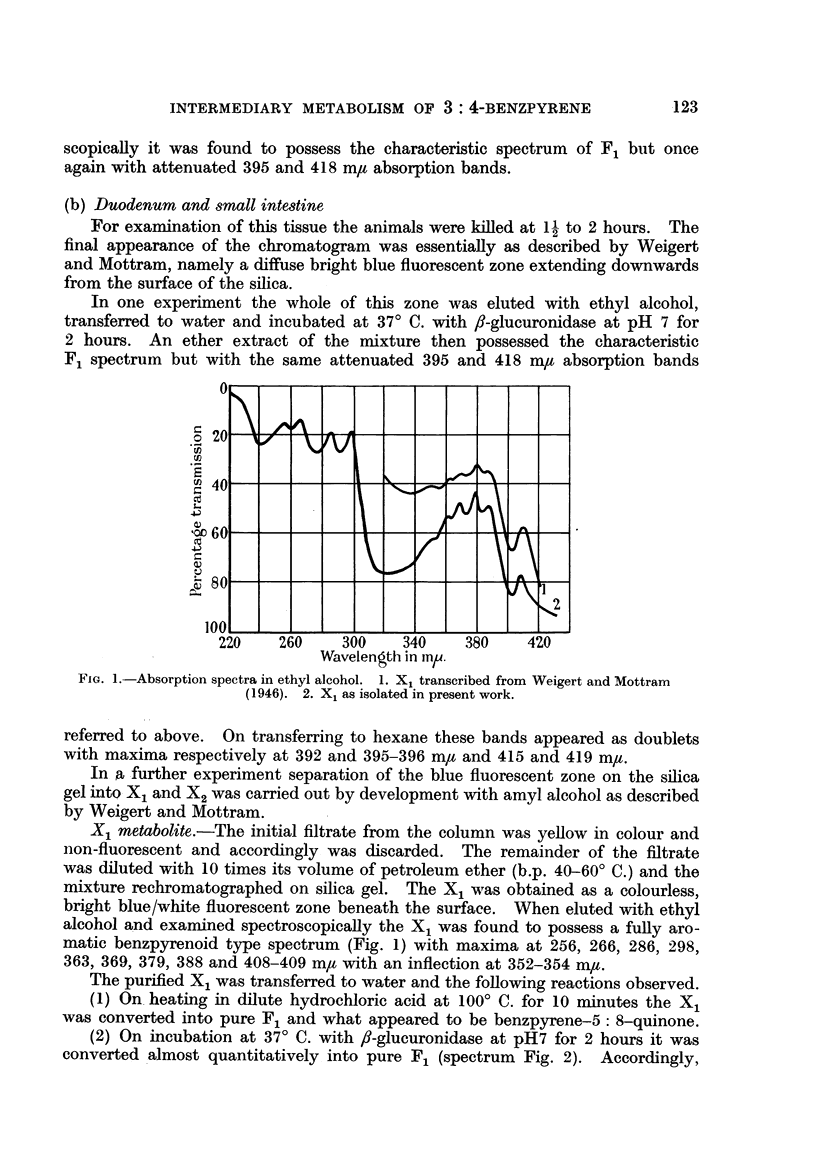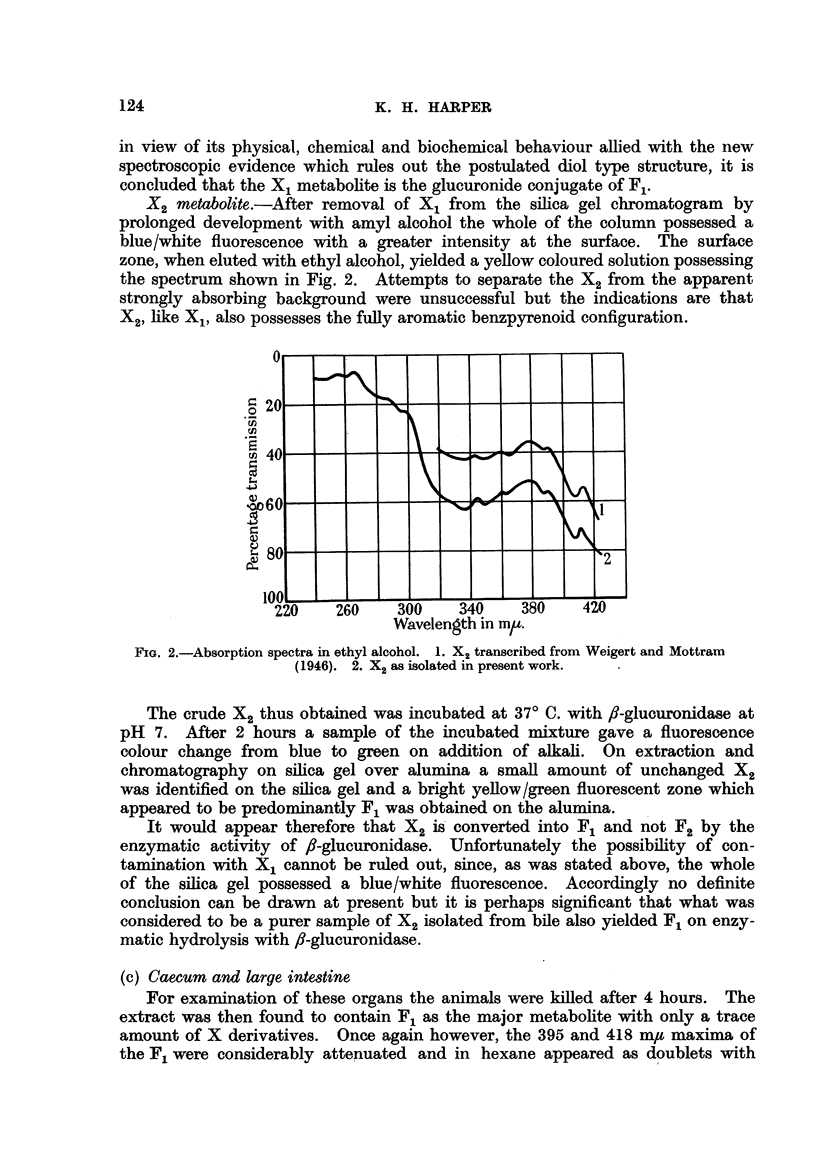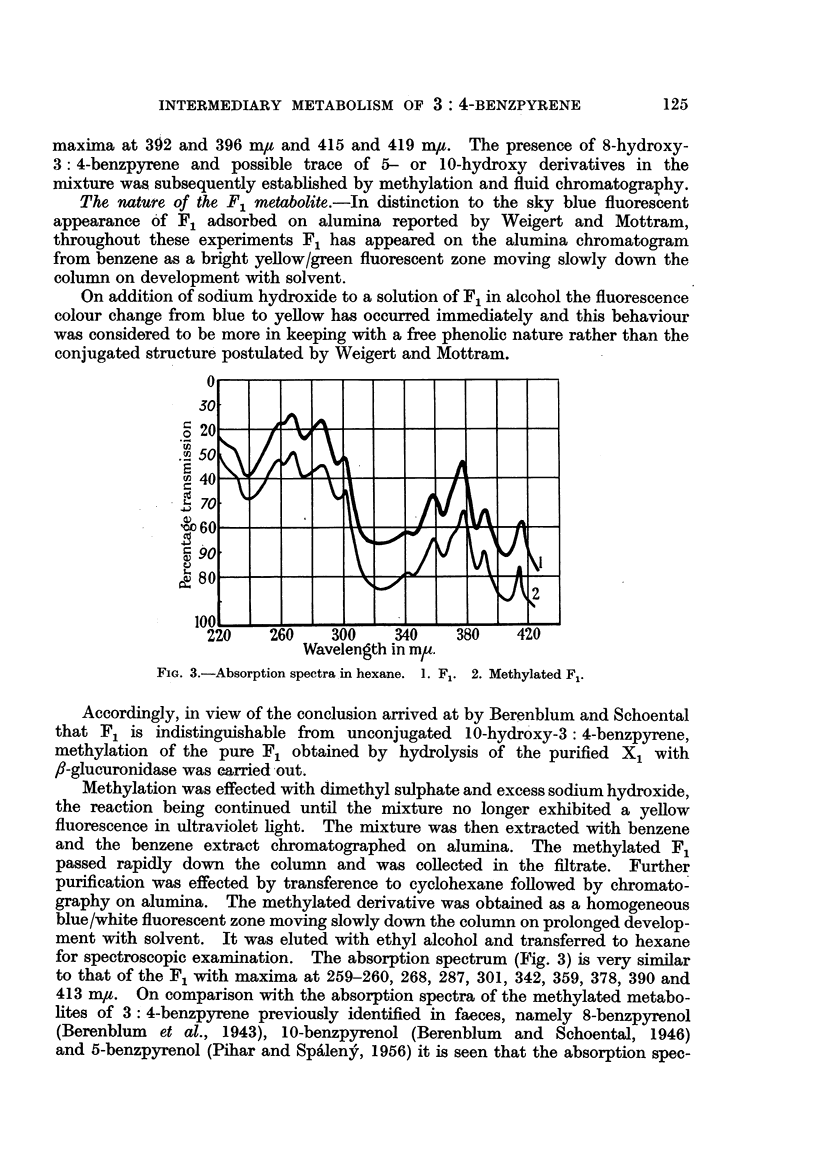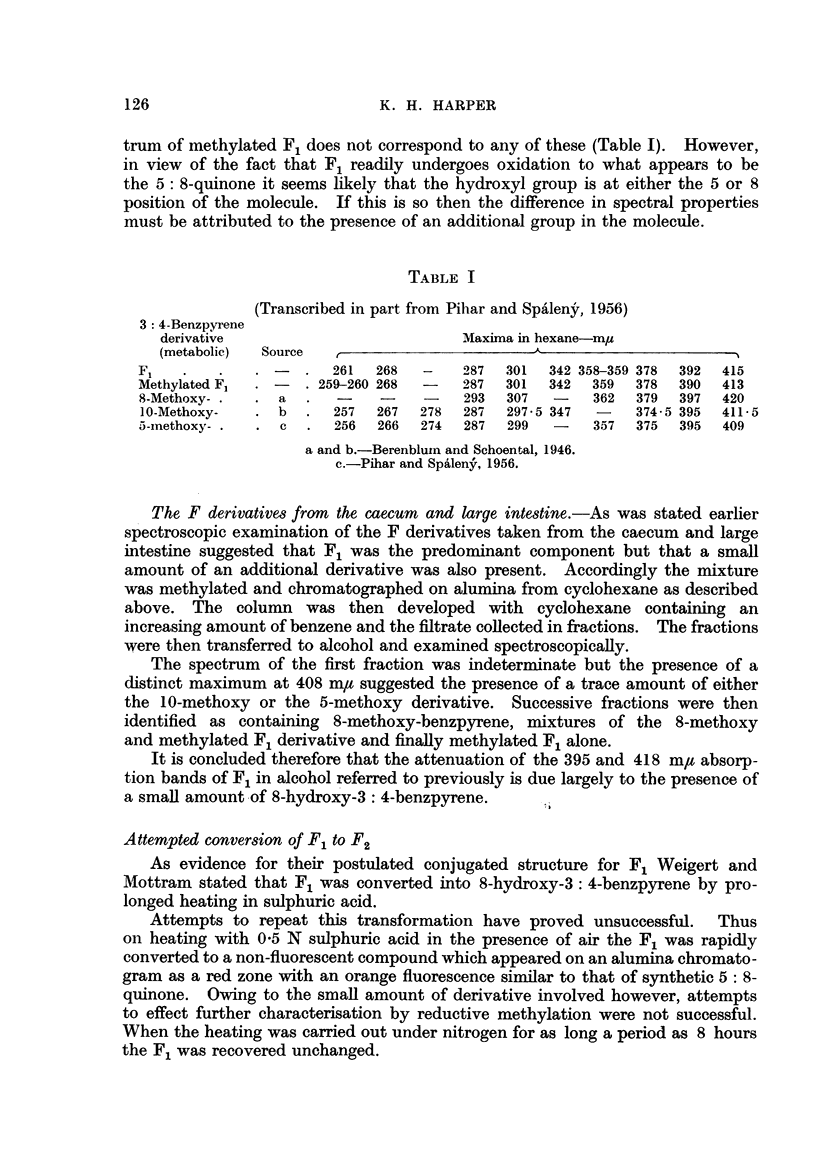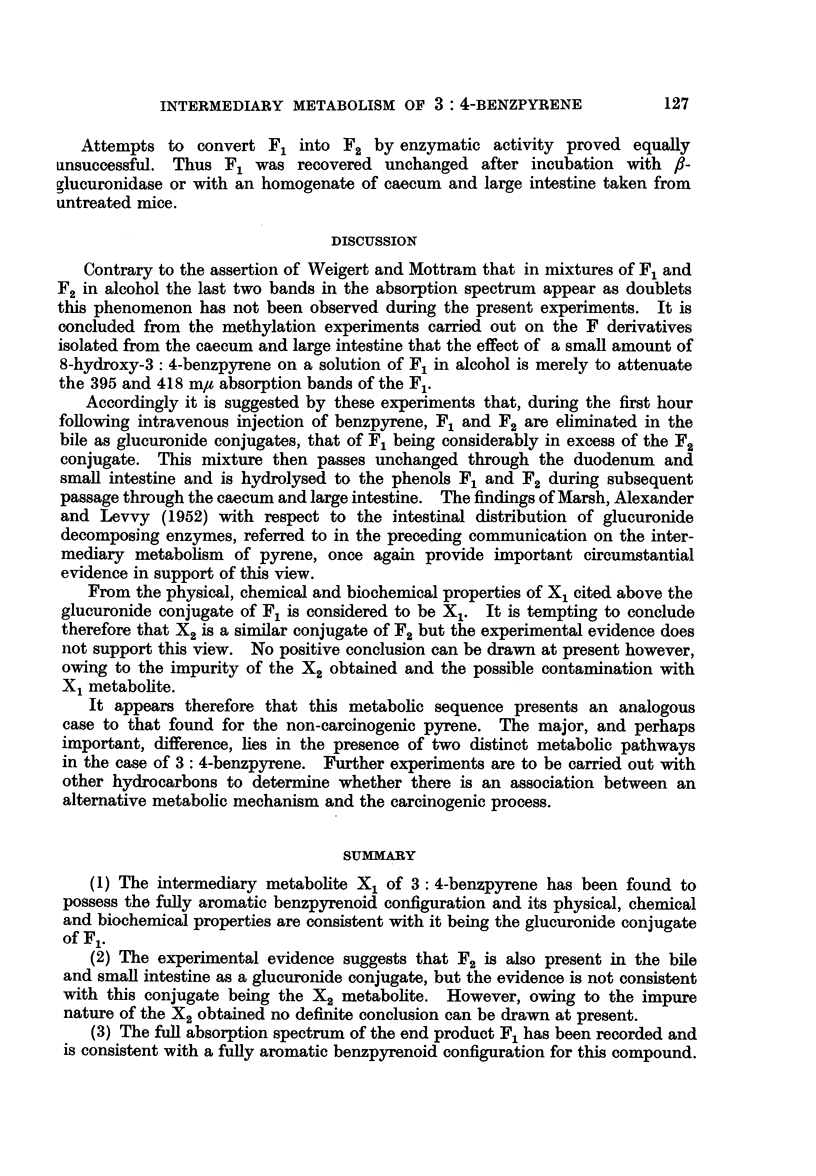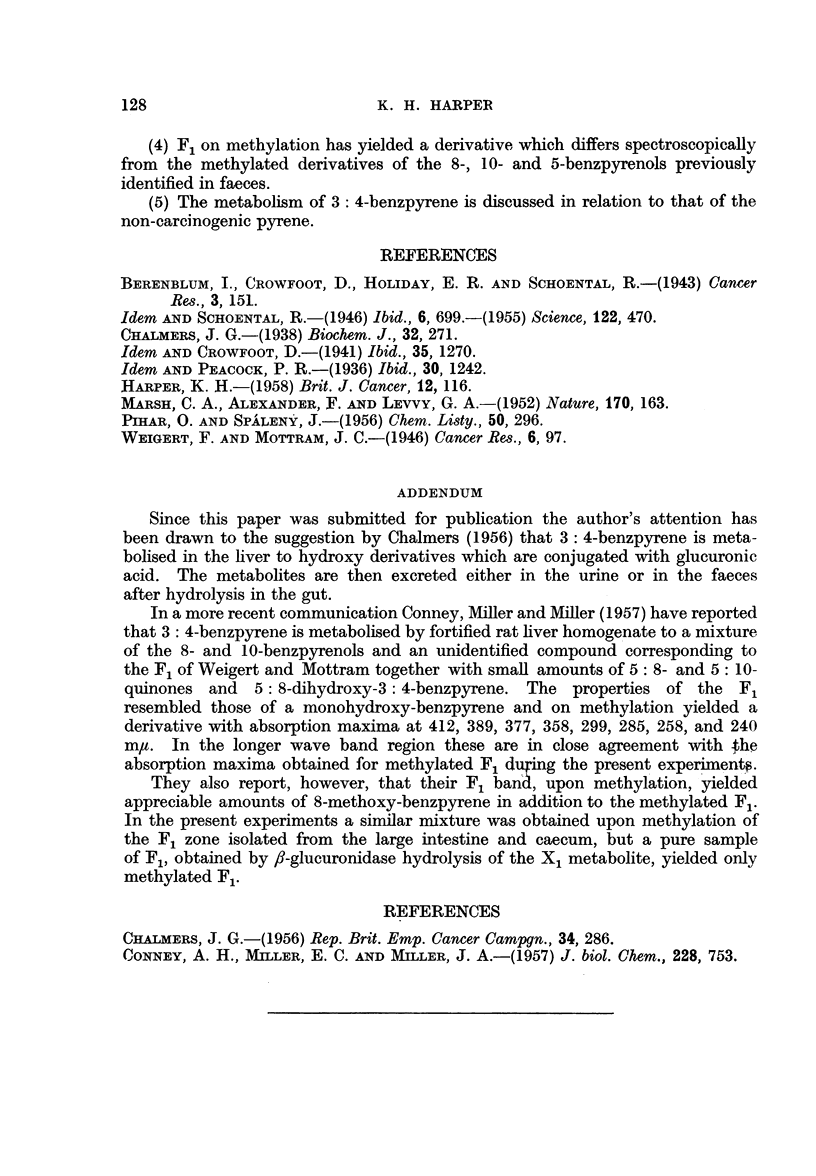# The Intermediary Metabolism of 3:4-Benzpyrene

**DOI:** 10.1038/bjc.1958.16

**Published:** 1958-03

**Authors:** K. H. Harper


### THE INTERMEDIARY METABOLISM OF 3 : 4-BENZPYRENE

K. H. HARPER

*From the Department of Cancer Research, Mount Vernon Hospital and the Radium Institute, Northwood, Middlesex*

Received for publication December 12, 1957

FOLLOWING on from previous findings that 3 : 4-benzpyrene is metabolised to two distinct derivatives, BPX and BPF, which differ in chemical properties and fluorescence spectra (Chalmers and Peacock, 1936 ; Chalmers, 1938 ; Chalmers and Crowfoot, 1941), Weigert and Mottram (1946) succeeded in isolating four different metabolites symbolised as $X_1$, $X_2$, $F_1$ and $F_2$. The X derivatives only were extractable from the liver, bile and small intestine whereas the F derivatives were present as major metabolites in the large intestine and faeces. The F derivatives were also found when tissues containing the X metabolites were stored for any length of time. In view of the apparent metabolic transformation and chemical properties of the derivatives and by analogy with the known metabolic fate of the non-carcinogenic hydrocarbon, anthracene, they postulated the following oxidative mechanism.

The groups $R_1$ and $R_2$ were not identified but an acidic nature was postulated for them.

The metabolite $F_2$ proved to be identical with 8-hydroxy-3 : 4-benzpyrene previously identified as an end product in the faeces (Berenblum, Crowfoot, Holiday and Schoental, 1943).

This reasoning, however, has been criticised by Berenblum and Schoental (1955) who argue that the long wave systems of the absorption spectra of $X_1$ and $X_2$ are very similar to those of fully aromatic benzpyrene derivatives and that $F_1$ is indistinguishable from unconjugated 10-hydroxy-3 : 4-benzpyrene previously identified in faeces (Berenblum and Schoental, 1946).

In view of the finding that the metabolism of the non-carcinogenic hydrocarbon pyrene proceeds via tissue intermediates possessing the fully aromatic pyrenoid configuration (Harper, 1958) it seemed desirable therefore to re-examine the metabolism of 3 : 4-benzpyrene in the hope of resolving this controversy.

### MATERIALS AND METHODS

Mice of RIII, Strong A, C.B.A. and Swiss strains were used for these experiments. Each mouse received an intravenous injection of 0·5 c.c. of a colloidal dispersion of 3 : 4-benzpyrene containing 1 mg. per c.c. The animals were then killed at intervals of 1 to 4 hours depending upon the particular organ under investigation.

The duodenum and small intestine and the caecum and large intestine were subjected to an extraction procedure essentially that devised by Weigert and Mottram (1946) except that silica gel (100/200 mesh) was used in preference to silica (100/150 mesh) for chromatography. This modification was found to give a much higher development rate and cleaner products on elution with ethyl alcohol.

### RESULTS

### (a) *Gall bladder*

For examination of bile the animals were killed after 1 hour. The bright blue fluorescent bile was first extracted with ether but this removed only a small amount of fluorescent material. After acidification, however, an ether extract possessed a strong blue/white fluorescence and when examined spectroscopically was found to possess a fully aromatic benzpyrenoid type spectrum. It was obtained free from colouring matter by the method previously described for the 3-hydroxypyrene glucuronide (Harper, 1957), that is by evaporating a solution in slightly moist ether and washing the residue with several small volumes of benzene. The residue dissolved in ethyl alcohol possessed absorption maxima at 257, 267, 287, 298, 361, 379 and 412 m$\mu$ and in view of the absorption spectra obtained subsequently for purified $X_1$ and $X_2$ it was considered to contain a mixture of these two derivatives.

A solution of the mixture in water possessed an intense blue/white fluorescence which remained unchanged on the addition of alkali.

A sample, heated in dilute hydrochloric acid at 100° C. for 10 minutes, assumed a yellow colour and on extraction with benzene followed by chromatography on alumina yielded a bright yellow/green fluorescent zone near the surface and a reddish coloured, orange fluorescent zone moving slowly down the column. On elution with ethyl alcohol the yellow/green fluorescent zone yielded a blue fluorescent solution which spectroscopically appeared to be the $F_1$ of Weigert and Mottram. However, attenuation of the 395 and 418 m$\mu$ absorption bands suggested the presence of an additional substance. The orange fluorescent zone was similar in appearance and behaviour to synthetic benzpyrene-5 : 8-quinone but the small quantity available prevented complete characterisation.

A further sample of the mixture was incubated at 37° C. with $\beta$-glucuronidase (bacterial) at pH 7 for 2 hours. An ether extract of the incubated mixture was then colourless with a strong blue/white fluorescence. When examined spectroscopically it was found to possess the characteristic spectrum of $F_1$ but once again with attenuated 395 and 418 mμ absorption bands.

(b) *Duodenum and small intestine*

For examination of this tissue the animals were killed at 1½ to 2 hours. The final appearance of the chromatogram was essentially as described by Weigert and Mottram, namely a diffuse bright blue fluorescent zone extending downwards from the surface of the silica.

In one experiment the whole of this zone was eluted with ethyl alcohol, transferred to water and incubated at 37° C. with β-glucuronidase at pH 7 for 2 hours. An ether extract of the mixture then possessed the characteristic $F_1$ spectrum but with the same attenuated 395 and 418 mμ absorption bands

FIG. 1.—Absorption spectra in ethyl alcohol. 1. $X_1$ transcribed from Weigert and Mottram (1946). 2. $X_1$ as isolated in present work.

referred to above. On transferring to hexane these bands appeared as doublets with maxima respectively at 392 and 395–396 mμ and 415 and 419 mμ.

In a further experiment separation of the blue fluorescent zone on the silica gel into $X_1$ and $X_2$ was carried out by development with amyl alcohol as described by Weigert and Mottram.

*$X_1$ metabolite*.—The initial filtrate from the column was yellow in colour and non-fluorescent and accordingly was discarded. The remainder of the filtrate was diluted with 10 times its volume of petroleum ether (b.p. 40–60° C.) and the mixture rechromatographed on silica gel. The $X_1$ was obtained as a colourless, bright blue/white fluorescent zone beneath the surface. When eluted with ethyl alcohol and examined spectroscopically the $X_1$ was found to possess a fully aromatic benzpyrenoid type spectrum (Fig. 1) with maxima at 256, 266, 286, 298, 363, 369, 379, 388 and 408–409 mμ with an inflection at 352–354 mμ.

The purified $X_1$ was transferred to water and the following reactions observed.

(1) On heating in dilute hydrochloric acid at 100° C. for 10 minutes the $X_1$ was converted into pure $F_1$ and what appeared to be benzpyrene–5 : 8–quinone.

(2) On incubation at 37° C. with β-glucuronidase at pH7 for 2 hours it was converted almost quantitatively into pure $F_1$ (spectrum Fig. 2). Accordingly, in view of its physical, chemical and biochemical behaviour allied with the new spectroscopic evidence which rules out the postulated diol type structure, it is concluded that the $X_1$ metabolite is the glucuronide conjugate of $F_1$.

*$X_2$ metabolite.*—After removal of $X_1$ from the silica gel chromatogram by prolonged development with amyl alcohol the whole of the column possessed a blue/white fluorescence with a greater intensity at the surface. The surface zone, when eluted with ethyl alcohol, yielded a yellow coloured solution possessing the spectrum shown in Fig. 2. Attempts to separate the $X_2$ from the apparent strongly absorbing background were unsuccessful but the indications are that $X_2$, like $X_1$, also possesses the fully aromatic benzpyrenoid configuration.

Fig. 2.—Absorption spectra in ethyl alcohol. 1. $X_2$ transcribed from Weigert and Mottram (1946). 2. $X_2$ as isolated in present work.

The crude $X_2$ thus obtained was incubated at 37° C. with β-glucuronidase at pH 7. After 2 hours a sample of the incubated mixture gave a fluorescence colour change from blue to green on addition of alkali. On extraction and chromatography on silica gel over alumina a small amount of unchanged $X_2$ was identified on the silica gel and a bright yellow/green fluorescent zone which appeared to be predominantly $F_1$ was obtained on the alumina.

It would appear therefore that $X_2$ is converted into $F_1$ and not $F_2$ by the enzymatic activity of β-glucuronidase. Unfortunately the possibility of contamination with $X_1$ cannot be ruled out, since, as was stated above, the whole of the silica gel possessed a blue/white fluorescence. Accordingly no definite conclusion can be drawn at present but it is perhaps significant that what was considered to be a purer sample of $X_2$ isolated from bile also yielded $F_1$ on enzymatic hydrolysis with β-glucuronidase.

(c) *Caecum and large intestine*

For examination of these organs the animals were killed after 4 hours. The extract was then found to contain $F_1$ as the major metabolite with only a trace amount of X derivatives. Once again however, the 395 and 418 mμ maxima of the $F_1$ were considerably attenuated and in hexane appeared as doublets with maxima at 392 and 396 mμ and 415 and 419 mμ. The presence of 8-hydroxy-3 : 4-benzpyrene and possible trace of 5- or 10-hydroxy derivatives in the mixture was subsequently established by methylation and fluid chromatography.

*The nature of the $F_1$ metabolite.*—In distinction to the sky blue fluorescent appearance of $F_1$ adsorbed on alumina reported by Weigert and Mottram, throughout these experiments $F_1$ has appeared on the alumina chromatogram from benzene as a bright yellow/green fluorescent zone moving slowly down the column on development with solvent.

On addition of sodium hydroxide to a solution of $F_1$ in alcohol the fluorescence colour change from blue to yellow has occurred immediately and this behaviour was considered to be more in keeping with a free phenolic nature rather than the conjugated structure postulated by Weigert and Mottram.

FIG. 3.—Absorption spectra in hexane. 1. $F_1$. 2. Methylated $F_1$.

Accordingly, in view of the conclusion arrived at by Berenblum and Schoental that $F_1$ is indistinguishable from unconjugated 10-hydroxy-3 : 4-benzpyrene, methylation of the pure $F_1$ obtained by hydrolysis of the purified $X_1$ with $\beta$-glucuronidase was carried out.

Methylation was effected with dimethyl sulphate and excess sodium hydroxide, the reaction being continued until the mixture no longer exhibited a yellow fluorescence in ultraviolet light. The mixture was then extracted with benzene and the benzene extract chromatographed on alumina. The methylated $F_1$ passed rapidly down the column and was collected in the filtrate. Further purification was effected by transference to cyclohexane followed by chromatography on alumina. The methylated derivative was obtained as a homogeneous blue/white fluorescent zone moving slowly down the column on prolonged development with solvent. It was eluted with ethyl alcohol and transferred to hexane for spectroscopic examination. The absorption spectrum (Fig. 3) is very similar to that of the $F_1$ with maxima at 259–260, 268, 287, 301, 342, 359, 378, 390 and 413 mμ. On comparison with the absorption spectra of the methylated metabolites of 3 : 4-benzpyrene previously identified in faeces, namely 8-benzpyrenol (Berenblum *et al.*, 1943), 10-benzpyrenol (Berenblum and Schoental, 1946) and 5-benzpyrenol (Pihar and Spálený, 1956) it is seen that the absorption spectrum of methylated $F_1$ does not correspond to any of these (Table I). However, in view of the fact that $F_1$ readily undergoes oxidation to what appears to be the 5 : 8-quinone it seems likely that the hydroxyl group is at either the 5 or 8 position of the molecule. If this is so then the difference in spectral properties must be attributed to the presence of an additional group in the molecule.

TABLE I

(Transcribed in part from Pihar and Spálený, 1956)

| 3 : 4-Benzpyrene derivative (metabolic) | Source | Maxima in hexane—mμ | | | | | | | | | |
|---|---|---|---|---|---|---|---|---|---|---|---|
| $F_1$ | — | 261 | 268 | – | 287 | 301 | 342 | 358–359 | 378 | 392 | 415 |
| Methylated $F_1$ | — | 259–260 | 268 | — | 287 | 301 | 342 | 359 | 378 | 390 | 413 |
| 8-Methoxy- | a | — | — | — | 293 | 307 | — | 362 | 379 | 397 | 420 |
| 10-Methoxy- | b | 257 | 267 | 278 | 287 | 297·5 | 347 | — | 374·5 | 395 | 411·5 |
| 5-methoxy- | c | 256 | 266 | 274 | 287 | 299 | — | 357 | 375 | 395 | 409 |

a and b.—Berenblum and Schoental, 1946.
c.—Pihar and Spálený, 1956.

*The F derivatives from the caecum and large intestine.*—As was stated earlier spectroscopic examination of the F derivatives taken from the caecum and large intestine suggested that $F_1$ was the predominant component but that a small amount of an additional derivative was also present. Accordingly the mixture was methylated and chromatographed on alumina from cyclohexane as described above. The column was then developed with cyclohexane containing an increasing amount of benzene and the filtrate collected in fractions. The fractions were then transferred to alcohol and examined spectroscopically.

The spectrum of the first fraction was indeterminate but the presence of a distinct maximum at 408 mμ suggested the presence of a trace amount of either the 10-methoxy or the 5-methoxy derivative. Successive fractions were then identified as containing 8-methoxy-benzpyrene, mixtures of the 8-methoxy and methylated $F_1$ derivative and finally methylated $F_1$ alone.

It is concluded therefore that the attenuation of the 395 and 418 mμ absorption bands of $F_1$ in alcohol referred to previously is due largely to the presence of a small amount of 8-hydroxy-3 : 4-benzpyrene.

*Attempted conversion of $F_1$ to $F_2$*

As evidence for their postulated conjugated structure for $F_1$ Weigert and Mottram stated that $F_1$ was converted into 8-hydroxy-3 : 4-benzpyrene by prolonged heating in sulphuric acid.

Attempts to repeat this transformation have proved unsuccessful. Thus on heating with 0·5 N sulphuric acid in the presence of air the $F_1$ was rapidly converted to a non-fluorescent compound which appeared on an alumina chromatogram as a red zone with an orange fluorescence similar to that of synthetic 5 : 8-quinone. Owing to the small amount of derivative involved however, attempts to effect further characterisation by reductive methylation were not successful. When the heating was carried out under nitrogen for as long a period as 8 hours the $F_1$ was recovered unchanged.

Attempts to convert $F_1$ into $F_2$ by enzymatic activity proved equally unsuccessful. Thus $F_1$ was recovered unchanged after incubation with $\beta$-glucuronidase or with an homogenate of caecum and large intestine taken from untreated mice.

### DISCUSSION

Contrary to the assertion of Weigert and Mottram that in mixtures of $F_1$ and $F_2$ in alcohol the last two bands in the absorption spectrum appear as doublets this phenomenon has not been observed during the present experiments. It is concluded from the methylation experiments carried out on the F derivatives isolated from the caecum and large intestine that the effect of a small amount of 8-hydroxy-3 : 4-benzpyrene on a solution of $F_1$ in alcohol is merely to attenuate the 395 and 418 m$\mu$ absorption bands of the $F_1$.

Accordingly it is suggested by these experiments that, during the first hour following intravenous injection of benzpyrene, $F_1$ and $F_2$ are eliminated in the bile as glucuronide conjugates, that of $F_1$ being considerably in excess of the $F_2$ conjugate. This mixture then passes unchanged through the duodenum and small intestine and is hydrolysed to the phenols $F_1$ and $F_2$ during subsequent passage through the caecum and large intestine. The findings of Marsh, Alexander and Levvy (1952) with respect to the intestinal distribution of glucuronide decomposing enzymes, referred to in the preceding communication on the intermediary metabolism of pyrene, once again provide important circumstantial evidence in support of this view.

From the physical, chemical and biochemical properties of $X_1$ cited above the glucuronide conjugate of $F_1$ is considered to be $X_1$. It is tempting to conclude therefore that $X_2$ is a similar conjugate of $F_2$ but the experimental evidence does not support this view. No positive conclusion can be drawn at present however, owing to the impurity of the $X_2$ obtained and the possible contamination with $X_1$ metabolite.

It appears therefore that this metabolic sequence presents an analogous case to that found for the non-carcinogenic pyrene. The major, and perhaps important, difference, lies in the presence of two distinct metabolic pathways in the case of 3 : 4-benzpyrene. Further experiments are to be carried out with other hydrocarbons to determine whether there is an association between an alternative metabolic mechanism and the carcinogenic process.

### SUMMARY

(1) The intermediary metabolite $X_1$ of 3 : 4-benzpyrene has been found to possess the fully aromatic benzpyrenoid configuration and its physical, chemical and biochemical properties are consistent with it being the glucuronide conjugate of $F_1$.

(2) The experimental evidence suggests that $F_2$ is also present in the bile and small intestine as a glucuronide conjugate, but the evidence is not consistent with this conjugate being the $X_2$ metabolite. However, owing to the impure nature of the $X_2$ obtained no definite conclusion can be drawn at present.

(3) The full absorption spectrum of the end product $F_1$ has been recorded and is consistent with a fully aromatic benzpyrenoid configuration for this compound.

(4) $F_1$ on methylation has yielded a derivative which differs spectroscopically from the methylated derivatives of the 8-, 10- and 5-benzpyrenols previously identified in faeces.

(5) The metabolism of 3 : 4-benzpyrene is discussed in relation to that of the non-carcinogenic pyrene.

### REFERENCES

BERENBLUM, I., CROWFOOT, D., HOLIDAY, E. R. AND SCHOENTAL, R.—(1943) *Cancer Res.*, **3**, 151.
*Idem* AND SCHOENTAL, R.—(1946) *Ibid.*, **6**, 699.—(1955) *Science*, **122**, 470.
CHALMERS, J. G.—(1938) *Biochem. J.*, **32**, 271.
*Idem* AND CROWFOOT, D.—(1941) *Ibid.*, **35**, 1270.
*Idem* AND PEACOCK, P. R.—(1936) *Ibid.*, **30**, 1242.
HARPER, K. H.—(1958) *Brit. J. Cancer*, **12**, 116.
MARSH, C. A., ALEXANDER, F. AND LEVVY, G. A.—(1952) *Nature*, **170**, 163.
PIHAR, O. AND SPÁLENÝ, J.—(1956) *Chem. Listy.*, **50**, 296.
WEIGERT, F. AND MOTTRAM, J. C.—(1946) *Cancer Res.*, **6**, 97.

### ADDENDUM

Since this paper was submitted for publication the author's attention has been drawn to the suggestion by Chalmers (1956) that 3 : 4-benzpyrene is metabolised in the liver to hydroxy derivatives which are conjugated with glucuronic acid. The metabolites are then excreted either in the urine or in the faeces after hydrolysis in the gut.

In a more recent communication Conney, Miller and Miller (1957) have reported that 3 : 4-benzpyrene is metabolised by fortified rat liver homogenate to a mixture of the 8- and 10-benzpyrenols and an unidentified compound corresponding to the $F_1$ of Weigert and Mottram together with small amounts of 5 : 8- and 5 : 10-quinones and 5 : 8-dihydroxy-3 : 4-benzpyrene. The properties of the $F_1$ resembled those of a monohydroxy-benzpyrene and on methylation yielded a derivative with absorption maxima at 412, 389, 377, 358, 299, 285, 258, and 240 m$\mu$. In the longer wave band region these are in close agreement with the absorption maxima obtained for methylated $F_1$ during the present experiments.

They also report, however, that their $F_1$ band, upon methylation, yielded appreciable amounts of 8-methoxy-benzpyrene in addition to the methylated $F_1$. In the present experiments a similar mixture was obtained upon methylation of the $F_1$ zone isolated from the large intestine and caecum, but a pure sample of $F_1$, obtained by $\beta$-glucuronidase hydrolysis of the $X_1$ metabolite, yielded only methylated $F_1$.

### REFERENCES

CHALMERS, J. G.—(1956) *Rep. Brit. Emp. Cancer Campgn.*, **34**, 286.
CONNEY, A. H., MILLER, E. C. AND MILLER, J. A.—(1957) *J. biol. Chem.*, **228**, 753.